# Release and Establishment of the Parasitoid *Diachasmimorpha kraussii* Against the Tephritid Fruit Fly *Bactrocera latifrons* in Hawaii

**DOI:** 10.1673/031.013.0701

**Published:** 2013-01-31

**Authors:** Aimé H. Bokonon-Ganta, Grant T. McQuate, Russell H. Messing, Eric B. Jang

**Affiliations:** 1 Plant Protection and Quarantine Service, Direction of Agriculture (SPVCP/DAGRI), 01 B.P. 58, Oganla, PortoNovo, Bénin; 2 USDA-ARS, U.S. Pacific Basin Agricultural Research Center (USDA-ARS, PBARC), 64 Nowelo Street, HiIo, HI 96720, USA; 3 University of Hawaii at Manoa, Department of Plant and Environmental Protection Sciences, Kauai Agricultural Research Center (KARC), Kapaa, HI 96746, USA

**Keywords:** biological control, Braconidae, Diptera, Hymenoptera, *Solanum torvum*, Tephritidae

## Abstract

*Diachasmimorpha kraussii* (Fullaway) (Hymenoptera: Braconidae) was first released against *Bactrocera latifrons* (Hendel) (Diptera: Tephritidae) in Hawaii in March 2003. Over a three month period, eight releases, totaling 7,696 females and 3,968 males, were made in a turkeyberry, *Solanum torvum* Swartz (Solanales: Solanaceae) patch known to have a well established *B. latifrons* population. The establishment of *D. kraussii* was assessed through fruit collections conducted over a three-year period beyond the last release. *D. kraussii* was recovered 2 weeks, 31 months, and 39 months after the last parasitoid release, with collections not only from the release site, but also from a control site about 5.0 km distance from the release site. Recovery from fruit collections three years after the last parasitoid release confirmed that *D. kraussii* had become established in Hawaii. Parasitism rates were low, only 1.0–1.4%, compared to rates of 2.8–8.7% for the earlier established egg-larval parasitoid, *Fopius arisanus* (Sonan).

## Introduction

*Bactrocera latifrons* (Hendel) (Diptera: Tephritidae) is the fourth and most recent invasive tephritid fruit fly species of economic importance to become established in Hawaii. It was first reported in Hawaii in 1983 (Vargas and Nishida 1985a, b) and has since been found to have a host range in Hawaii of at least 15 plant species in the families Solanaceae and Cucurbitaceae (Liquido et al. 1994).

A number of classical biological control programs were conducted against the first three invasive tephritid fruit fly species established in Hawaii *(Bactrocera cucurbitae* (Coquillett), *Ceratitis capitata* (Wiedemann), and *B. dorsalis* (Hendel)) (e.g., Clausen 1956; Clausen et al. 1965; Wharton 1989), but no classical biological control programs have been conducted against *B. latifrons* since its accidental introduction in Hawaii. (Messing and Ramadan 2000). Preliminary data indicated that parasitism rates of *B. latifrons* by extant parasitoids were low, with parasitism rates of *Fopius arisanus* (Sonan), the primary parasitoid found, only about 5.2% (Bokonon-Ganta et al. 2007). Therefore, this study introduced *Diachasmimorpha kraussii* (Fullaway) (Hymenoptera: Braconidae), a new parasitoid species with the potential to improve biological control of *B. latifrons*.

*D. kraussii* is an Australian parasitoid of *Bactrocera tryoni* (Froggatt) and several other endemic tephritids (Rungrojwanisch and Walter 2000). *D. kraussii* was previously introduced into Hawaii in 1949, when it was targeted against *B. dorsalis*, and over 25,000 individuals were released from 1950 to 1954. However, it failed to become established. Subsequent laboratory studies in Hawaii showed that *D. kraussii* eggs were consistently encapsulated by *B. dorsalis* larvae (Messing and Ramadan 2000), but that *D. kraussii* successfully parasitized all three instars of *C. capitata* and *B. latifrons* (Messing and Ramadan 2000). Furthermore, tests with non-target tephritids in Hawaii showed minimal risk of *D. kraussii* to nontarget species (Duan and Messing 2000). *D. kraussii* is known to reproduce under a variety of climatic conditions (Sime et al. 2006). In addition, it rapidly searches for and handles hosts, and aggressively eliminates supernumeraries during the larval stage (Wang and Messing 2004). The potential effectiveness of *D. kraussii* against *B. latifrons*, combined with minimal risk of adverse effects on non-targets, led to the approval by federal authorities and the Hawaii State Board of Agriculture for it to be released in fields. Presented in this paper are the results of efforts to establish *D. kraussii* against a *B. latifrons* population established in thickets of a wild solanaceous weed, turkeyberry, *Solanum torvum* Swartz (Solanales: Solanaceae), a plant known to be a good host of *B. latifrons* (Liquido et al. 1994; Bokonon-Ganta et al. 2007; McQuate et al. 2007).

## Materials and Methods

### Rearing

In April 1996, a cohort of 434 *D. kraussii* (74% female) was shipped to the quarantine facility of the Hawaii Department of Agriculture in Honolulu, Hawaii, from a laboratory colony in Queensland, Australia. From this cohort, a small colony (∼500 males and 500 females) of *D. kraussii* was maintained in quarantine for about three years until approval to release was given in 1998. *D. kraussii* was then removed from quarantine and reared in insectaries on the islands of Kauai and Oahu, using *B. latifrons* as a rearing host. *B. latifrons* larvae were provided by the USDA-ARS Pacific Basin Agricultural Research Center, Honolulu, Hawaii. Adult parasitoids were maintained in the insectary at 28 ± 2° C, 60–80% RH, with a 12:12 L:D photoperiod, in 25 × 25 × 25 cm wooden screened cages, and given water and spun pure honey ad libitum. Subsequent parasitoid colonies were initiated using oviposition units made of 9 cm diameter plastic petri dishes with tight-fitting lids covered with organdy, through which the wasps could oviposit (Wong and Ramadan 1992). These units were packed with ∼600 third instar *B. latifrons* host larvae and artificial rearing medium, and were exposed to the parasitoids for about 24 hrs. After exposure, the contents of the oviposition units were transferred to incubation units made of plastic cups (9 cm diameter, 5 cm depth) with 150 g fresh wheat diet (Tanaka et al. 1969) as a supplemental food for young larvae to continue development. Each cup was placed in a 4 L plastic container (20 cm diameter, 20 cm depth) with a hole (10 cm diameter) cut in the lid of the container and replaced with a fine mesh screen. A 1-cm layer of fine vermiculite was added to the bottom to serve as a substrate for pupation. This vermiculite was kept moist to prevent pupal desiccation. Ten days later, fruit fly puparia were sieved from the vermiculite, counted, and placed in clean rearing cages for emergence of adult parasitoids, which were fed with water and fine drops of pure spun honey on the top-side of the rearing cages.

### Packaging and transport

Adult *D. kraussii* (3–6 days old, both males and females) were collected from the rearing cages at the Kauai Agricultural Research Center and the Honolulu laboratory on Oahu by aspiration into 20 cm3 plastic vials, each containing a strip of filter paper with a drop of pure spun honey. Each vial held an average of 25 individuals (range: 12–31). The vials were closed with a gauze cover. If necessary (e.g., when an early morning release was planned), they were stored overnight at 6 ± 2° C before being transported in insulated boxes to the release site. All releases were done within one to two hours after boxes were picked up from the air cargo station at the airport in Kahului, Maui, or two to three hours after packaging (unless overnight storage was needed).

### Release site

Although turkeyberry is known to be a good host of *B. latifrons* in Hawaii (Liquido et al. 1994; Bokonon-Ganta et al. 2007; McQuate et al. 2007), relatively accessible moderate-sized turkeryberry populations are widely scattered in Hawaii, making it logistically too challenging to attempt parasitoid releases at multiple sites while also having comparable nearby control sites. However, two wellestablished turkeyberry patches known to support persistent *B. latifrons* populations (McQuate and Peck 2001; McQuate et al. 2004, 2007) were in close enough proximity to serve as release and control sites for the *D. kraussii* release trial.

**Figure 1.**
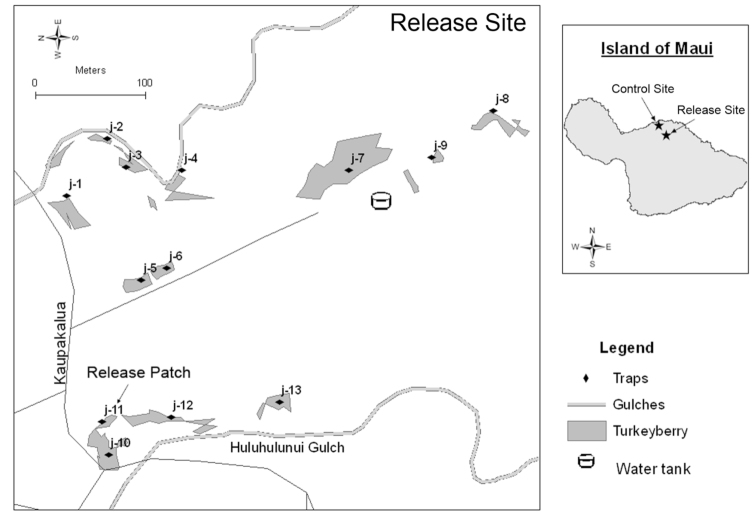
Locations of the *Diachasmimorpha kraussii* release site and control site, with indications of the relative location of *Solanum torvum* patches where fruit collections were made and alpha-ionol + cade oil traps were deployed. The *S. torvum* patch where all *D. kraussii* releases were made (“release patch”) is indicated with an arrow. High quality figures are available online.

The release patch at the release site (j-11 in Figure 1), where all releases were done, was a 93.0 m^2^ well-developed thicket of turkeyberry plants in the Huluhulunui Gulch, adjacent to Kaupakalua Road, north of Kokomo, on the island of Maui in Hawaii, U.S.A. (20° 53′ 5.6796″ lat, -156° 17′, 56.209). Additional patches of turkeyberry were present in the vicinity of the release site. Patch size averaged about 457 m^2^, ranging from about 93 m^2^ to 2190 m^2^. Relative size and locations of all patches are illustrated in Figure 1. These patches considered together are hereafter referred to as the release site. The control site for this study was in Haiku, about 5 km downstream from the release site, and it also had many patches of turkeyberry. Both sites are on the northern side of Maui (which is also the windward side) and included small valleys, with elevation over the sites varying about 33 m in the vicinity of the release site and about 18 m at the control site. The release site, with a higher elevation than the control site (388 and 158 m respectively), typically has a higher average rainfall. Rainfall totaled 226 cm at the release site versus 189 cm at the control site from 25 February 2003 to 24 August 2004 (2.9 versus 2.4 cm/week). Rainfall totals were not obtained after 24 August 2004, because lengthened times between fruit collections did not permit the requisite regular servicing of the weather station.

**Table 1.**
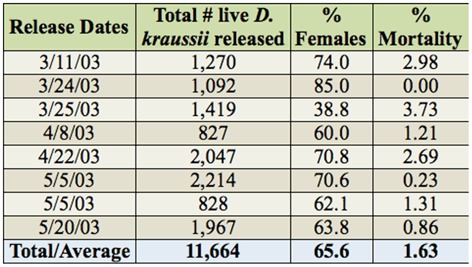
Summary of releases of *Diachasmimorpha kraussii* against *Bactrocera latifrons*. Percentage mortality refers to numbers of live wasps packaged before shipment.

### Release methods

All releases were conducted between 11 March 2003 and 20 May 2003 in the thicket of turkeyberry plants at the release patch (j-11 in Figure 1). Releases were not made at the other turkeyberry patches shown on the map of the release site (Figure 1). *D. kraussii* individuals released were from about the 85^th^ generation from the initial cohort received in Honolulu. The numbers of parasitoids released on each of the eight shipment dates are presented in Table 1. Dates and times of releases, as well as weather conditions at the times of releases, are summarized in Table 2. The turkeyberry patch where all eight *D. kraussii* releases were made (“release patch”) is indicated in Figure 1. Because of the logistics required to transport the parasitoids to a different island for release, release times varied among dates, ranging from 08:00 to 18:15 (see Table 2). Parasitoids were released from the plastic vials onto turkeyberry foliage.

**Table 2.**
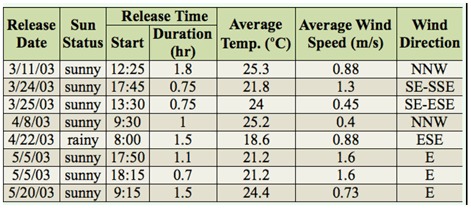
Summary of times and weather conditions during the releases of *Diachasmimorpha kraussii* against *Bactrocera latifrons*.

### Monitoring adult *B. latifrons* populations

Adult *B. latifrons* populations were monitored at 13 separate turkeyberry patches at the release site, and 10 separate turkeyberry patches at the control site, through the use of Jackson traps baited with 2.0 mL alpha-ionol (“latilure”: 4-(2,6,6-trimethyl-2-cyclohexen-lyl)-3-buten-2-ol, obtained from Bedoukian Research, Inc., http://www.bedoukian.com/) and 1.0 mL rectified cade oil (Penta Manufacturing, http://www.pentamfg.com/) deployed on separate 3.8 cm long × 1.0 cm diameter cotton wicks, each enclosed in separate plastic baskets hung from the middle of the trap hanger. The bait is attractive to sexually mature male *B. latifrons* adults, with males exhibiting 50% and 75% of peak response at 7 and 14 days old, respectively (McQuate et al. 2008). A single trap was placed in each turkeyberry patch. At the release site, one trap was placed in the patch where the *D. kraussii* releases were made, with additional traps placed in three other turkeyberry patches in Huluhulunui Gulch (j10, j-12, j-13 in Figure 1), at four sites along a nearby stream channel (j-1, j-2, j-3, and j-4 in Figure 1), and at five sites along a ridge between Huluhulunui Gulch and the nearby stream channel (j-5, j-6, j-7, j-8, and j-9 in Figure 1). All of the traps at the control site, and 12 of the 13 traps at the release site were deployed on 14 January 2003. The thirteenth trap at the release site (j-13) was set on 11 March 2003. Traps were serviced every two weeks from the date of deployment until trapping ceased on 7 September 2004. All wicks were replaced with freshly charged wicks every eight weeks. After 7 September 2004, continued presence of an adult *B. latifrons* population was inferred through fruit infestation data (see below).

### Monitoring *D. kraussii* establishment

In order to assess whether *D. kraussii* became established, regular fruit collections of yellow (ripe) turkeyberry fruits were initiated on 14 January 2003 (control site) and 11 February 2003 (release site), eight and four weeks, respectively, before the first *D. kraussii* release, and were continued every two weeks until 10 August 2004 (over one year after the last release). Green (unripe) turkeyberry fruits were found not suitable for infestation by *B. latifrons* (McQuate et al 2007) and were not collected. Care was taken that no more than one half of the ripe fruits present were collected at a time in order to ensure that the potential for local establishment of the parasitoid would not be adversely impacted. The collections began before the release of *D. kraussii* in order to record other parasitoids that are associated with *B. latifrons* that might be adversely impacted as well. Following 10 August 2004, fruits were collected quarterly for more than three years after the last *D. kraussii* release (1 December 2004; 16 March 2005; 15 June 2005; 22 September 2005; 7 December 2005; 14 February 2006; 8 March 2006; and 14 August 2006). Fruits were counted, weighed, and then placed in screened containers, which held sand as a pupation medium. The sand was sieved through a strainer weekly for four successive weeks in order to recover pupariating larvae and puparia, which were then held individually in separate containers for recovery of emerging flies and parasitoids. All species of flies and parasitoids that were recovered were identified and counted. Parasitoid identifications were initially made in the laboratories of Aimé H. Bokonon-Ganta and Grant T. McQuate based on locally established keys (unpublished) and prior experience, and were subsequently confirmed by Mohsen Ramadan, Hawaii State Department of Agriculture.

## Results

### Parasitoid releases

A total of 11,664 adult *D. kraussii* individuals, of which 65% were female, were released between 11 March 2003 and 20 May 2003. Mortality during transport was very low (∼1.63% Table 1). Parasitoids found dead at the time of release were not included in the reported totals.

### Monitoring adult *B. latifrons* populations

**Figure 2.**
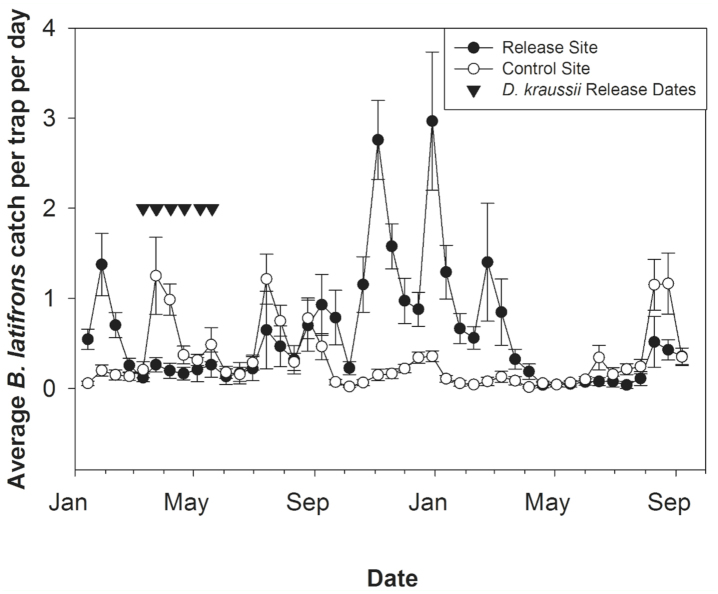
Average (± SEM) male *Bactrocera latifrons* catch per trap per day at the release site and at the control site over the course of the study, with dates of releases of *Diachasmimorpha kraussii* indicated. High quality figures are available online.

Male lure-trap catches at both sites varied over time (Figure 2). At the control site, over the 18 months of the study where the adult field populations were monitored, the *B. latifrons* populations were typically stable, with the highest recorded trap catch being 1.2 flies per trap per day. At the release site, *B. latifrons* populations were highly variable, with 7.9 flies/trap/day being the highest trap catch at any trap site. The maximum catch at other trap sites was recorded on 30 December 2003, with this date also having the highest trap catch averaged over all traps for any date (3.0 flies/trap/day). *B. latifrons* populations were typically highest in late summer to early winter, and lowest in late winter to early spring, when there was a lapse of flowering and fruiting of turkeyberry. *B. latifrons* populations were low, but present, over the course of the *D. kraussii* releases at both the release patch and at the other turkeyberry patches at the release site (Figure 2).

### Turkeyberry abundance

**Figure 3.**
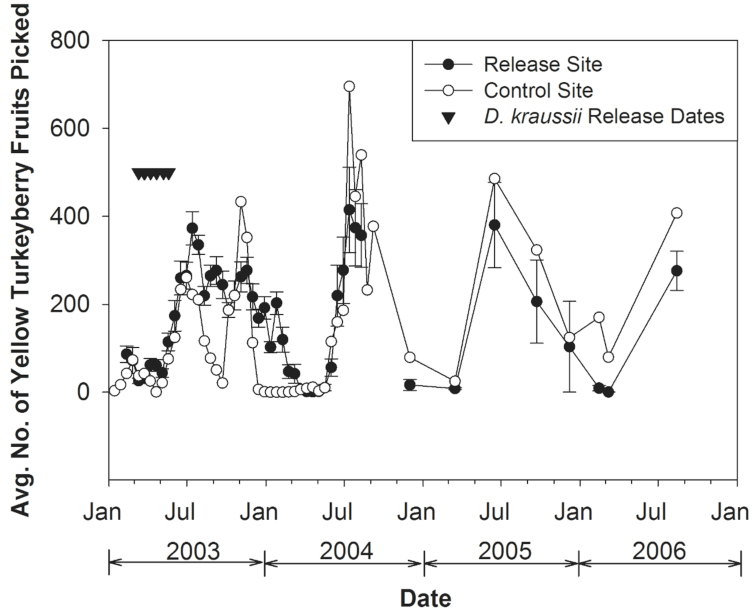
Average (± SEM) numbers of yellow *Solanum torvum* fruits collected at the release site and at the control site over the course of the study, with dates of releases of *Diachasmimorpha kraussii* indicated. High quality figures are available online.

The number of fruits collected was highly variable with time for both sites (Figure 3), with cycles of fruit abundance varying between sites. At the release patch, the low point for ripe fruit recovery was 11 March 2003 (12 fruits), but ripe fruits were still present over the course of the *D. kraussii* releases. This date also had the lowest number of collected yellow fruits averaged over all collection sites at the release site (25.9 fruits; see Figure 3). In 2004, the low point for ripe fruit recovery at the release patch was AprilMay, when no ripe fruits were collected. This period was a low point in ripe fruit collections throughout the release site with only 0.85 fruits recorded (on April 19), the lowest 2004 ripe fruit collection averaged over all patches (Figure 3). Some ripe fruits were present at other sites. Collection numbers gradually increased beginning in June 2004. At the control site, the low point of ripe fruit availability came on 21 April 2003 (an average of 0.6 ripe fruits per patch), but came much earlier in 2004 (26 January) (0 ripe fruits). At the release patch, peak ripe turkeyberry recovery in 2003 and 2004 occurred on 30 June (517 fruits) and on 26 January (170 fruits) respectively. Peak ripe fruit recovery, averaged over all patches at the release site, however, came a bit later in each year (372.9 fruits on 14 July 2003, and 414.7 fruits on 13 July 2004) (Figure 3). At the control site, peak ripe turkeyberry recovery in 2003 and 2004 occurred on 4 November (an average of 433.2 fruits/site) and 13 July (an average of 695.5 fruits/site), respectively. Although turkeyberry abundance varied considerably over the subsequent quarterly ripe fruit collections, at least some ripe fruits were recovered at each collection time at both the release site and at the control site (Figure 3).

**Table 3.**
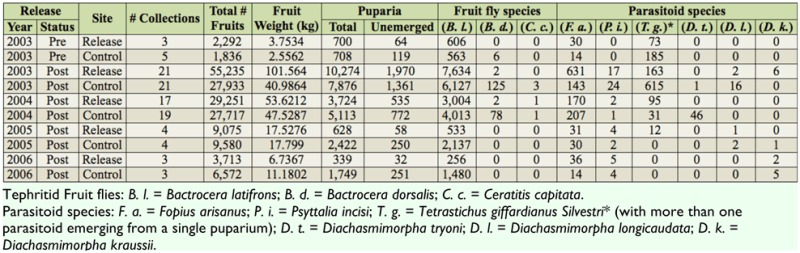
Summary of numbers of yellow *Solanum torvum* fruit pre-release (status: pre) and post-release (status: post) collections, numbers and weights of fruits collected, and numbers of fruit fly individuals *(Bactrocera latifrons, B. dorsalis*, and *Ceratitis capitata)* and six parasitoid species recovered from 2003 to 2006 in Kaupakalua (release site) and Haiku (control site), both on Maui, Hawaii. Numbers of parasitoids + numbers of flies + number of unemerged puparia may not equal the total number of puparia because multiple parasitoids may emerge from a single puparium, as routinely occurs with *Testrastichus giffardianus*.

### Pupal recovery

**Figure 4.**
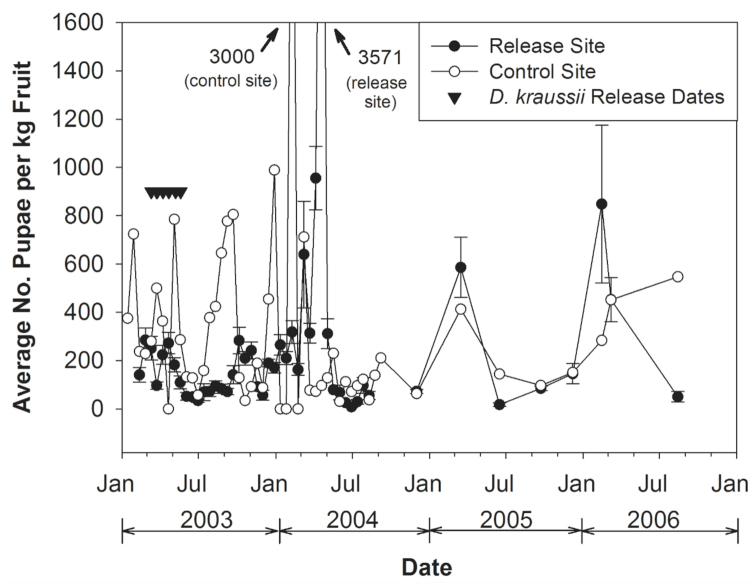
Average rates of infestation of yellow *Solanum torvum* fruits by *Bactrocera latifrons* (puparia per kg fruit) at the release site and at the control site over the course of the study, with dates of releases of *Diachasmimorpha kraussii* indicated. High quality figures are available online.

The pupal recovery rate was also highly variable with time for both sites. At the release patch, the peaks of pupal recoveries/kg fruit were on 11 March in 2003 (447 puparia/kg fruit; the date of the first *D. kraussii* release) and 23 February in 2004 (1149 puparia/kg fruit). Peak pupal recovery averaged over all turkeyberry patches at the release site came a bit earlier in 2003 (285 puparia/kg ripe fruit on 25 February) and a bit later in 2004 (717 puparia/kg ripe fruit on 5 April) (Figure 4). At the control site, there were six peaks of pupal recoveries that exceeded 700 puparia/kg fruit between 28 January 2003 and 10 August 2004, with the highest (3,000 puparia/kg) occurring on 9 February 2004 (a time of low fruit abundance). Although pupal recovery varied considerably over the subsequent quarterly ripe fruit collections, at least some *B. latifrons* were recovered at each collection time at both the release site and at the control site (Figure 3).

### Monitoring *D. kraussii *establishment

Two weeks after the first release, adult female *D. kraussii* were observed searching on turkeyberry leaves and fruits at the release site. Further evidence of *D. kraussii* presence came through larval recoveries from ripe fruit collections. Table 3 shows the total number of collections, the total number of ripe turkeyberry fruits collected, and the recoveries of *B. latifrons*, other tephritid fruit fly species, *D. kraussii*, and other parasitoid species. The six *D. kraussii* reported in Table 3 from 2003 were recovered on 3 June, two weeks after the last *D. kraussii* release, with two females and one male recovered from patch j-7, one female recovered from patch j-8, and two females recovered from patch j-9. No *D. kraussii* were recovered again until 7 December 2005, when one male was recovered from the control site, located about 5.0 km from the release site. Because of the 7 December recovery, it was apparent that additional collections were needed to further verify establishment. Consequently, three additional collections were made in 2006. In the third collection (15 August), one male and one female *D. kraussii* were recovered from patch j-8 at the release site, and three males and two females were recovered from the control site. Based on the 2005 and 2006 recoveries, it was concluded that *D. kraussii* was established at multiple sites as a parasitoid of *B. latifrons*. The parasitism rate, though, is quite low at both sites. Using data from the 2006 collections where *D. kraussii* was recovered at both treatment and control sites, the parasitism rate of *D. kraussii* (calculated from the number of *D. kraussii* relative to the total number of pupae recovered, because *B. latifrons* was the only tephritid fruit fly recovered in the 2006 fruit collections) was 1.0% (5/494 *B. latifrons* puparia) at the control site and 1.4% (2/138 *B. latifrons* puparia) at patch 8 (j8 in Figure 1) at the release site. This parasitism rate was comparable to that of *Psyttalia incisi* (Silvestri) at both sites (0.8% (4/494 *B. latifrons* puparia) at the control site and 1.4% (2/138 *B. latifrons* puparia) at patch 8 at the release site). However, the rate was considerably less than that of *F. arisanus* (2.8% (14/494 *B. latifrons* puparia) at the control site and 8.7% (12/138 *B. latifrons* puparia) at site 8 at the release site).

## Discussion

This paper documents the first inoculative release and first successful establishment of *D. kraussii* for biological control of *B. latifrons* in Hawaii. The success was achieved on turkeyberry, which is known to be a good host and is present as an invasive weed over extended areas. Although a number of other *B. latifrons* hosts are known (e.g., Liquido et al. 1994), they would be more difficult to use for testing of parasitoid establishment because of limited plant populations, either as wild or cultivated host plants.

Classical biological control literature is divided over whether it is a better strategy to release a large number of parasitoids in a few places (e.g., Beirne 1975; Cameron et al. 1993; Grevstad 1999) or a small number of insects in many places (e.g., Campbell 1976; Memmot et al. 1998) to increase the probability of establishment. In the present study, over 10,000 *D. kraussii* adults were released over eight separate occasions, which is a large number in reference to the threshold of 1,000 insects recommended by Hopper and Roush (1993) as necessary to ensure establishment of introduced parasitoids. The number of insects released was considerably smaller than the 75,881 *D. kraussii* released against *Ceratitis capitata* in Israel, where successful establishment was also achieved (Argov and Gazit 2008). However, the number of insects released in the present study was larger than the number of 1,694 female and 212 male *Diachasmimorpha longicaudata* (Ashmead) released in Taiwan, which resulted in the successful recovery of the wasp (Yao 1989).

Generally, an exotic species is considered established if adults are recorded for at least one year after release at a given site (DeBach and Bartlett 1964). Even though *D. kraussii* was recovered in small numbers, the insects were consistently recovered two weeks, 31 months, and 39 months after the last release, demonstrating that the insect completed its life cycle under local conditions, thus documenting its establishment in Hawaii. Our data showed that the parasitoid was established in Hawaii, with rates of parasitism not exceeding 1.4%. The low rate of parasitism in the present study was consistent with results of inoculative releases of other braconid parasitoids against tephritid fruit flies, including the release of *F. arisanus* against *Ceratitis capitata* on *Coffea arabica* L. farms in Costa Rica, which resulted in the recovery of the wasp with a low impact (Wharton et al. 1981). Similarly, in Australia, *F. arisanus* was introduced and established on the native *B. tryoni* in 1962, but it reportedly had only a negligible effect (Quimio and Walter 2001). Yao (1989) reported successful recoveries with rates of parasitism variable between 0.6 and 9.1% following the inoculative release of *D. longicaudata* in Taiwan. Higher parasitism rates (12.37%) were also reported for *D. kraussii* following the larger releases made in Israel against *C. capitata* (Argov and Gazit 2008).

The low level of parasitism by *D. kraussii* reported in the present study could be because the wasps had been mass-reared on artificial diet, and did not get any previous experience on turkeyberry, which might also have influenced their searching efficiency. Another possible explanation for the low level of parasitism is that turkeyberry may not be a preferred host habitat for *D. kraussii* even if suitable fruit fly hosts are abundant. Alternatively, it is possible that the level of parasitism reported here underestimates the real parasitism rate because (1) fruit sampling may have caused a considerable reduction in the site density of fly larvae that could have been available for the parasitoids and resulted in low estimates of subsequent parasitism (Wong et al. 1991; Sivinski et al. 1996), or (2) some of the parasitoids established on *B. latifrons* infesting turkeyberry fruits on the ground (either freshly fallen or older, rotting fruits), which were not sampled (Bokonon-Ganta et al. 2007). The closely related *D. longicaudata* has been found to attack larvae in fallen fruit on the ground (Purcell et al. 1994). In that study, numbers of *D. longicaudata* per fruit and parasitism levels were highest in guavas that remained on the ground for 6–10 days.

Two years after the first release, *D. kraussii* was recovered 5 km from the release site. This dispersal might have been achieved through movement by the wind or transportation of fallen turkeyberry fruits by water or birds. This dispersal was in the range of 0.8 km/yr to 6.7 km/yr recorded by various authors for *Cotesia rubecula*, an introduced parasitoid of *Pieris rapae* (L.) (Puttler et al. 1970; McDonald and Kok 1992; Cameron and Walker 2002). However, because localities further away than the control site were not sampled, it was not possible to document a dispersal of *D. kraussii* further than 5 km. Rates of spread greater than 5 km per year were reported for several other microhymenoptera. For example, the parasitoid *Trioxys pallidus* Haliday, released against the walnut aphid in California, covered 130,000 km^2^ within two years (van den Bosch et al. 1970). Additionally, Cameron et al. (1989) reported two braconid parasitoids introduced for control of Lepidoptera in New Zealand. Among these: *Cotesia kazak* Telenga, *Pteromalus puparum* (L.), and *Diadromus collaris* (Gravenhorst) spread 100 km/yr, 110 km/yr, and 105 km/yr, respectively. Other examples of rapidly dispersing microhymenoptera include the encyrtid parasitoids *Apoanagyrus* (*Epidinocarsis*) *lopezi* De Santis, which covered 100 km per season (Herren et al. 1987), and *Gyranusoidea tebygi* Noyes, which was reported to cover 100 km/year (Neuenschwander et al. 1994).

The observed low level of parasitism by *D. kraussii* over the period extending three years after the last release suggests the need to reassess the status of establishment continuously in order to determine whether the low level of parasitism observed increases with time and starts to have significant impact on *B. latifrons* populations. Beyond making future assessments at the sites used in this study, additional releases could be tried under alternative conditions that may be conducive to more successful establishment, such as making releases at times of high *B. latifrons* population levels (McQuate et al. 2007), releasing in other *B. latifrons* host plant areas, or making releases in localities in Africa where *B. latifrons* has recently colonized (Mwatawala et al. 2007) and other parasitoid species may not be present. Alternatively, it may be that enhanced biological control of *B. latifrons* may better be achieved through augmentative releases of another, already established, parasitoid species, such as *F. arisanus*, which naturally established itself as the predominant parasitoid in Hawaii (Bokonon-Ganta et al. 2007; Aimé H. Bokonon-Ganta and Grant T. McQuate unpublished data).
